# The application of PET-CT to post-mastectomy regional radiation therapy using a deformable image registration

**DOI:** 10.1186/1748-717X-8-104

**Published:** 2013-04-29

**Authors:** Yu Sun Lee, Kyoung Ju Kim, Seung Do Ahn, Eun Kyung Choi, Jong Hoon Kim, Sang-wook Lee, Si Yeol Song, Sang Min Yoon, Young Seok Kim, Jin-hong Park, Byung Chul Cho, Su Ssan Kim

**Affiliations:** 1 Department of Radiation Oncology, Daejin Medical Center Bundang Jesaeng General Hospital, Seohyeon-dong, Sungnam-Si, Gyeonggi-Do, South Korea; 2 Department of Radiation Oncology, Kangnam Sacred Heart Hospital, Hallym University College of Medicine, Daerim 1-dong, Yeongdeungpo-gu, Seoul, South Korea; 3 Department of Radiation Oncology, Asan Medical Center, Ulsan University College of Medicine, Pungnap 2-dong, Songpa-gu, Seoul, South Korea

**Keywords:** Breast cancer, Deformable image registration (DIR), Radiotherapy, PET-CT

## Abstract

**Background:**

To evaluate the utility of the preoperative PET-CT using deformable image registration (DIR) in the treatment of patients with locally advanced breast cancer and to find appropriate radiotherapy technique for further adequate treatment of axillary nodal area.

**Methods:**

Sixty-five breast cancer patients who had level II, III axillary or supraclavicular lymph node metastasis on ^18^F-FDG PET-CT and received postoperative radiotherapy after modified radical mastectomy were enrolled. One radiation oncologist contoured normal organs (axillary vessels, clavicular head, coracoids process and humeral head) and involved lymph nodes on PET-CT and simulation CT slices. After contouring, deformable image registration of PET-CT on simulation CT was carried out. To evaluate the performance of the DIR, Dice similarity coefficient (DSC) and Center of mass (COM) were used. We created two plans, one was the historically designed three field plan and the other was the modified plan based on the location of axillary lymph node, and we compared the doses that irradiated the axillary lymph nodes.

**Results:**

The DSCs for axillary artery, axillary vein, clavicular head, coracoids process and humeral head were 0.43 ± 0.15, 0.39 ± 0.20, 0.85 ± 0.10, 0.72 ± 0.20 and 0.77 ± 0.20, respectively. The distances between the COMs of axillary artery, axillary vein, clavicular head, coracoids process and humeral head in simulation CT and from PET-CT were 13.0 ±7.1, 20.2 ± 11.2, 4.4 ± 6.3, 3.7 ± 6.7, and 9.5 ± 25.0 mm, respectively. In the historically designed plan, only 57.7% of level II lymph nodes received more than 95% of prescribed dose and the coverage was improved to 70.0% with the modified plan (p < 0.01). For level III lymph nodes, the volumes received more than 95% of prescribed dose were similar in both plans (96.8 % vs 97.9%, p = 0.35).

**Conclusion:**

Deformable image registration of PET-CT on simulation CT was helpful in the identification of the location of the preoperatively involved axillary lymph node. Historically designed three-field plan was not adequate to treat the axillary level II lymph node area. Novel treatment technique based on the location of axillary lymph node from PET-CT using DIR can result in more adequate coverage of nodal area.

## Introduction

Breast cancer is the second most common cancer in Korean women [1]. General management for patients with breast cancer is surgery. Breast conserving surgery and adjuvant radiotherapy is the standard treatment for early breast cancer and mastectomy with or without neoadjuvant chemotherapy for locally advanced breast cancer. Adjuvant radiotherapy after mastectomy is recommended for patients with large tumor (> 5 cm) or 4 or more axillary LN involvement [2]. Tumor cells that have remained in the locoregional lymphatics and chest wall may be the source of distant metastasis [3], so optimal irradiation to these areas is important to improve the tumor control. The target volume after mastectomy includes the chest wall and locoregional lymphatics. Postoperative adjuvant locoregional radiotherapy after mastectomy showed a survival advantage for women with lymph node positive breast cancer [4-7].

PET-CT is an emerging diagnostic modality in the management of most of the malignancies, including breast cancer. The sensitivity of PET or PET-CT in detecting axillary lymph node is only 63% [8] but hypermetabolism of an axillary lymph node in noninfectious condition is highly suggestive of metastasis [9,10]. There have been many efforts to use PET or PET-CT image for RT planning in various tumor sites [11-13]. Addition of PET image to RT planning can improve the accuracy of target delineation. Although PET-CT image acquired in the treatment position is ideal for target delineation, it is not possible in the treatment of breast cancer because the mainstream of treatment for breast cancer is surgery and radiotherapy is given postoperatively. Only preoperative PET-CT is available and this image cannot be directly used for RT planning. The possible method to incorporate the preoperative PET-CT image into RT planning is to use image registration tool. However, rigid image registration may be inadequate if there is a positional change, therefore deformable image registration (DIR) can be used. DIR has enormous possibilities for its application in the RT plan [14-17]. Recently, there has been commercial development of software for DIR.

In this study, we evaluated the utility of the preoperative PET-CT for radiotherapy using DIR and intended to find appropriate radiotherapy technique to treat axillary nodal area in a more adequate manner.

## Materials and methods

### Patient selection

From January 2009 to January 2011, 65 breast cancer patients who had level II, III axillary or supraclavicular lymph node metastasis on whole-body ^18^F-FDG PET-CT and received postoperative radiotherapy on chest wall and regional lymphatics after modified radical mastectomy (MRM) were enrolled for the present study.

The patient characteristics are shown in Table 1. Sixty-three patients (97%) had level II lymphadenopathy (LAP), 32 patients (49%) had level III LAP and 21 patients (32%) had supraclavicular LAP. Eighty nine percent of the patients (58/65) underwent percutaneous needle aspiration biopsy at the axillary nodal area before surgery and 90% of the patients (52/58) had nodal metastasis. Among the 6 patients, who showed negative biopsy finding, 5 of them were confirmed to have nodal metastasis after surgery. Fifty-four patients (83%) received neoadjuvant chemotherapy before surgery.

**Table 1:** Patient characteristics

	**No. of patients (%)**
Involved LN	
Level II only	28 (43%)
Level II + III	16 (25%)
Level II + III + SCN	14 (21%)
Level III + SCN	2 (3%)
Level II + SCN	5 (8%)
Biopsy positive, preoperatively	
Yes	52 (90%)
No	6 (10%)
Clinical Stage	
IIB	2 (3%)
IIIA	18 (28%)
IIIB	6 (9%)
IIIC	39 (60%)
The type of axillary surgery	
Sentinel node biopsy only	11 (16.9%)
ALND (level I + II)	36 (55.4%)
ALND (level I + II + III)	12 (18.5%)
ALND (level I + II) + SCND	6 (9.2%)

All patients underwent PET-CT at the time of diagnosis. PET-CT was performed on curved scanner table with both arms raised above the head. All patients underwent simulation CT after MRM and axillary surgery. The type of axillary surgery is shown in Table 1. Simulation CT scan was performed on sloped flat breast board with the ipsilateral arm raised above the head.

### Organ contouring and deformable image registration

**Figure 1:**
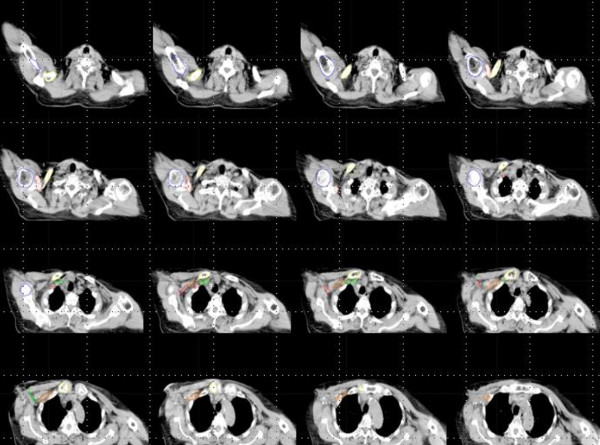
**Normal organs were contoured on simulation CT images.** Axillary vessels, clavicular head, coracoids process and humeral head were delineated.

**Figure 2:**
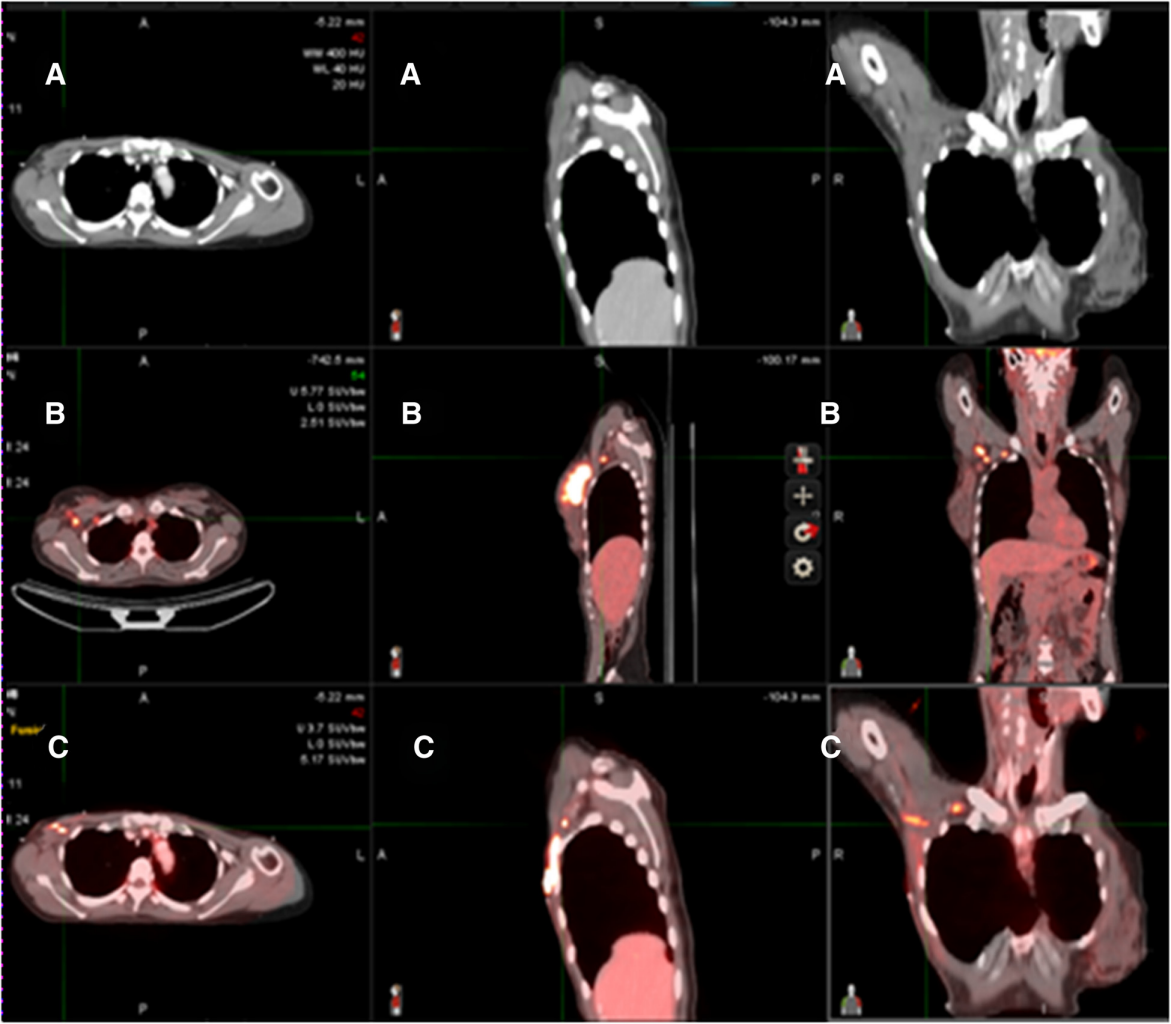
**Representative images after deformable image registration. A**: Simulation CT images. **B**: PET-CT images. **C**: Fusion images of deformed PET and simulation CT.

PET-CT and simulation CT images were transferred to radiation treatment planning system (Eclips, Varian Medical System). One radiation oncologist contoured normal organs and involved lymph nodes on PET-CT and simulation CT slices within planning system (Figure 1). The delineated normal organs were axillary vessels, clavicular head, coracoids process and humeral head. Target volumes were delineated on simulation CT according to RTOG breast cancer contouring atlas. Involved axillary lymph nodes were contoured only on PET-CT. After contouring, deformable image registration of PET-CT on simulation CT was carried out using commercially available intensity based deformable image registration software (MIM-Vista version 5.1, MIM software Inc, USA) (Figure 2). Subsequently, the normal organs and involved lymph nodes contoured on PET-CT were transferred to simulation CT. They were quantitatively compared with the contours delineated on simulation CT with respect to the correspondence. Two methods were employed to evaluate the performance of the DIR. One was Dice similarity coefficient (DSC) and the other was Center of mass (COM). The DSC measures the overlap between two structures and is represented from 0 (no overlap) to 1 (perfect overlap). It is defined by the formula; DSC = V_simCT_∩V_PET-CT_/{(V_simCT_ + V_PET-CT_)/2}, where V_simCT_ is the volume of contour delineated on simulation CT and V_PET-CT_ is the volume of contour transferred from PET-CT. We also measured the COM of each structures contoured on simulation CT and transferred from PET-CT and calculated the distance between the COMs of the structures in simulation CT and the ones transferred from PET-CT. For axillary vessels, the volumes that expanded 0.5 cm from axillary vessels were used in measuring DSC and the distance between the COMs because axillary vessels were very thin structures.

We also attempted to explore the clinical factors affecting DSC and the distance between the COMs. Pearson correlation coefficient was used to evaluate the association.

### Radiation technique

All the patients were treated on sloped flat breast board in supine position with the ipsilateral arm elevated above the head. The arm was immobilized with individualized mold.

**Figure 3:**
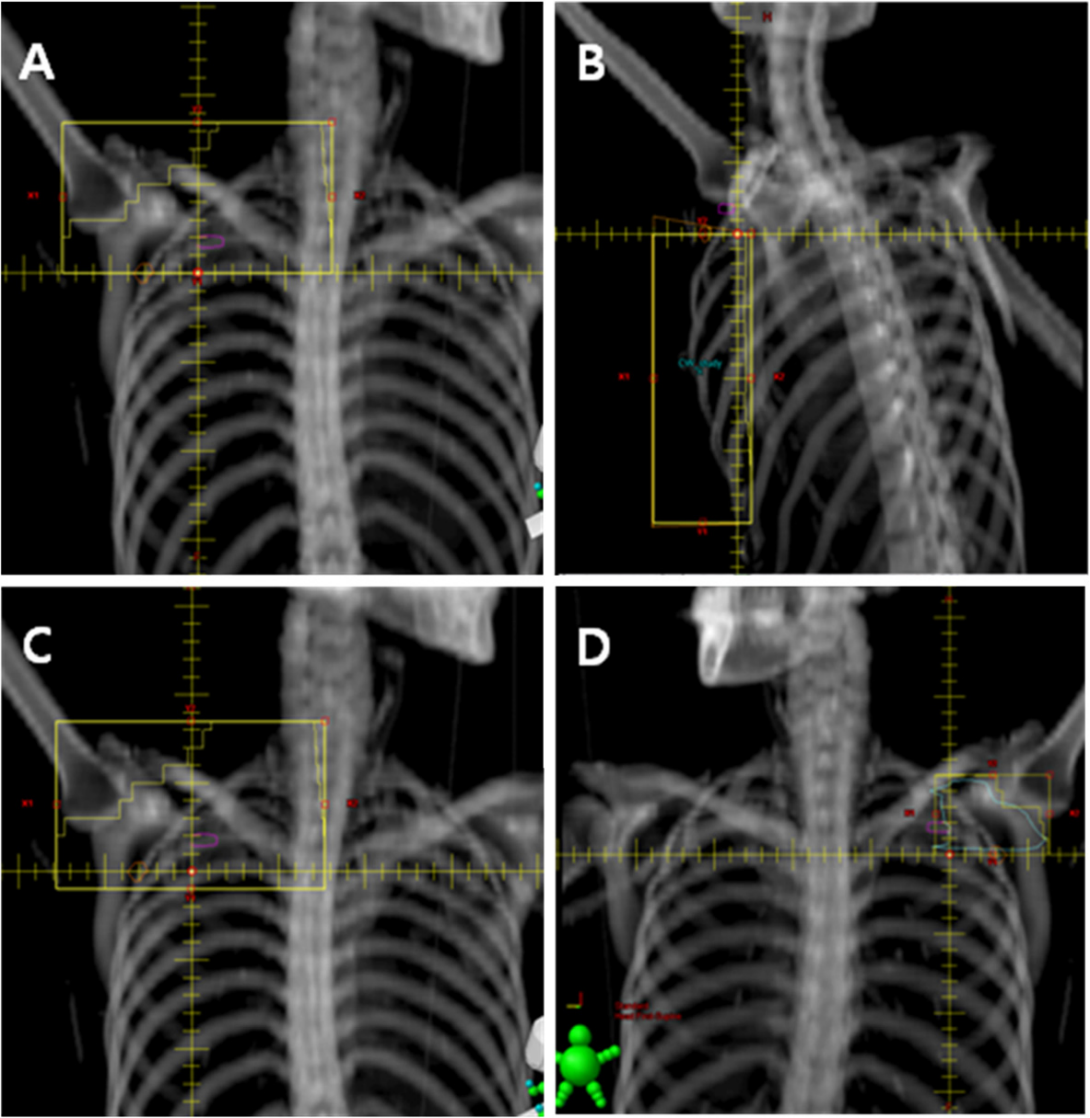
**Digitally reconstructed radiographs (DRR) image of each field. A**: Anterior field. It is the historically designed field. **B**: Tangential fields. They also follow the historically designed fields. **C**: Modified anterior field. It is 1 cm larger below the junction compared to the historical field. **D**: Posterior axillary boost beam. Sky blue line represents the area received the dose below 90% of the prescribed dose.

We created two plans, one was the historically designed plan and the other one was the modified plan, and compared the doses that irradiated the lymph nodes. In the historically designed plan, three beams were used for radiotherapy, one anterior beam to treat the level II, III and supraclavicular lymph nodes (Figure 3A) and two tangential beams to treat the chest wall (Figure 3B). The anterior beam was angulated to 5 degrees to reduce the dose to spinal cord and esophagus. Entire chest wall from midline to mid-axillary line was included in the tangential beams. Wedge was used to achieve uniform dose distribution. A single isocenter was employed to minimize matching problem between the anterior field and the tangent fields. The isocenter was located at the inferior border of the clavicular head according to the historical method. The prescribed dose was 50 Gy at 2 Gy per fraction. The dose to the anterior field was prescribed at a depth of 3 cm.

In the modified plan, beam arrangements were similar to the historical plan. Tangent beams were same to the historical plan. For the treatment of anterior field, 90% of the prescribed dose to anterior field was irradiated similarly to the historical plan but 10% of the dose was irradiated to modified anterior field. The modified anterior field was 1 cm larger below the junction compared to the historical field (Figure 3C). Accordingly, it overlapped 1 cm with the tangential field. The dose around the junction is always lower than the prescribed dose, so to compensate the under-dose, 10% of the prescribed dose was irradiated to 1 cm larger field below the junction. In the modified plan, posterior axillary boost (PAB) field was used (Figure 3D). After the doses delivered to the anterior field and the modified anterior field were calculated, the area received the dose below 90% of the prescribed dose was delineated and then PAB was performed on this area. The PAB dose was adjusted depending on the dose distribution.

### Statistical analysis

Pearson correlation coefficient was used for the analysis of an association between the variables. We analyzed the association between the clinical characteristics such as BMI (Body mass index) and the performance of DIR. Paired *t*-test was used to assess the differences between the variables. The tests were performed at the 0.05 level of significance. SPSS version 12.0 (SPSS Inc., Chicago, IL) was used for the statistical analysis.

## Results

### Evaluation of the performance of the DIR

**Figure 4:**
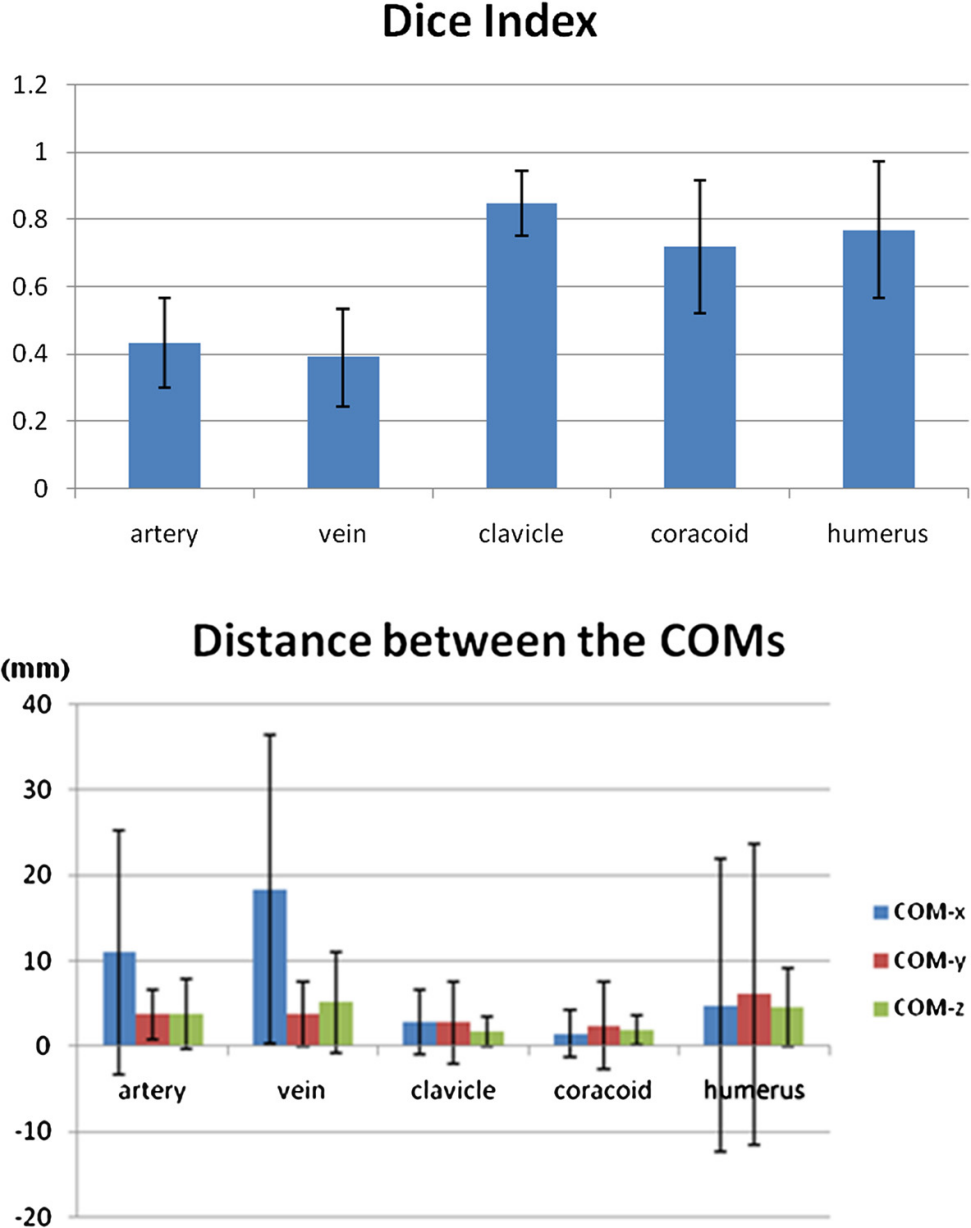
**The graphs show Dice similarity coefficient (DSC) and the distance between the Centers of mass (COMs) of structures.** The error bars represent 95% confidence interval.

The DSCs for axillary artery, axillary vein, clavicular head, coracoids process and humeral head were 0.43 ± 0.15 (Mean ± 95% confidence interval), 0.39 ± 0.20, 0.85 ± 0.10, 0.72 ± 0.20 and 0.77 ± 0.20, respectively (Table 2, Figure 4). The DSC value was highest for clavicle and lowest for axillary vessels.

**Table 2:** Mean DSC and the distance between the COMs of structures

	**Axillary artery**	**Axillary vein**	**Clavicle**	**Coracoid process**	**Humeral head**
DSC*					
mean ± 95% CI^†^	0.43 ± 0.15	0.39 ± 0.20	0.85 ± 0.10	0.72 ± 0.20	0.77 ± 0.20
Distance between the COMs^‡^ (lateral direction) (mm)					
mean ± 95% CI	11.0 ±7.6	18.4 ± 11.8	2.8 ± 3.7	1.4 ± 3.6	4.8 ± 17.0
Distance between the COMs (sup.-inf. direction) (mm)					
mean ± 95% CI	3.7 ± 2.2	3.7 ± 3.0	2.7 ± 4.8	2.4 ± 5.2	5.9 ± 17.5
Distance between the COMs (ant.-post. direction) (mm)					
mean ± 95% CI	3.7 ± 2.9	5.2 ± 4.1	1.7 ± 2.	1.9 ± 2.7	4.5 ± 7.4
Distance between the COMs (3 dimensional) (mm)					
mean ± 95% CI	13.0 ± 7.1	20.2 ± 11.2	4.4 ± 6.3	3.7 ± 6.7	9.5 ± 25.0

* Dice similarity coefficient, ^†^ Confidence interval, ^‡^ Centers of mass.

The distance between the COMs of axillary arteries in simulation CT and from PET-CT was 13.0 ± 7.1 mm (Mean ± 95% confidence interval). Those of the axillary vein, clavicular head, coracoids process and humeral head were 20.2 ± 11.2, 4.4 ± 6.3, 3.7 ± 6.7, and 9.5 ± 25.0 mm, respectively (Table 2, Figure 4). The distance between the COMs of structures was largest in axillary vessels and smallest in coracoids process. The distance of COMs for axillary vessels was largest in lateral direction.

We also evaluated clinical factors affecting DSC and the distance between the COMs. Because DSCs of axillary vessels were found to be the smallest among axillary vessels, clavicular head, coracoids process and humeral head, the correlations were analyzed between clinical factors and DSC and the distance between centers of axillary vessels. Any clinical features did not affect the DSC and the distance between centers of axillary vessels (Table 3).

**Table 3:** Correlation coefficient between the variables

	**Correlation coefficient**	**P value**
BMI* and ^†^DSC of axillary a.	0.74	0.56
BMI and distance between the COMs^‡^(axillary a.)	0.00	0.98
Level II volume and DSC of axillary a.	−0.11	0.41
Level II volume and distance between the COMs (axillary a.)	0.24	0.06
Initial weight and DSC of axillary a.	0.01	0.97
Initial weight and distance between the COMs (axillary a.)	0.18	0.14
Weight loss and DSC of axillary a.	−0.18	0.16
Weight loss and distance between the COMs (axillary a.)	0.24	0.06
BMI and DSC of axillary v.	0.05	0.70
BMI and distance between the COMs (axillary v.)	−0.22	0.08
Level II volume and DSC of axillary v.	−0.17	0.18
Level II volume and distance between the COMs (axillary v.)	0.16	0.21
Initial weight and DSC of axillary v.	−0.04	0.73
Initial weight and distance between the COMs (axillary v.)	−0.09	0.49
Weight loss and DSC of axillary v.	−0.16	0.19
Weight loss and distance between the COMs (axillary v.)	0.10	0.43

* Body mass index, ^†^ Dice similarity coefficient, ^‡^ Centers of mass.

### The locations of the involved axillary lymph nodes

**Figure 5:**
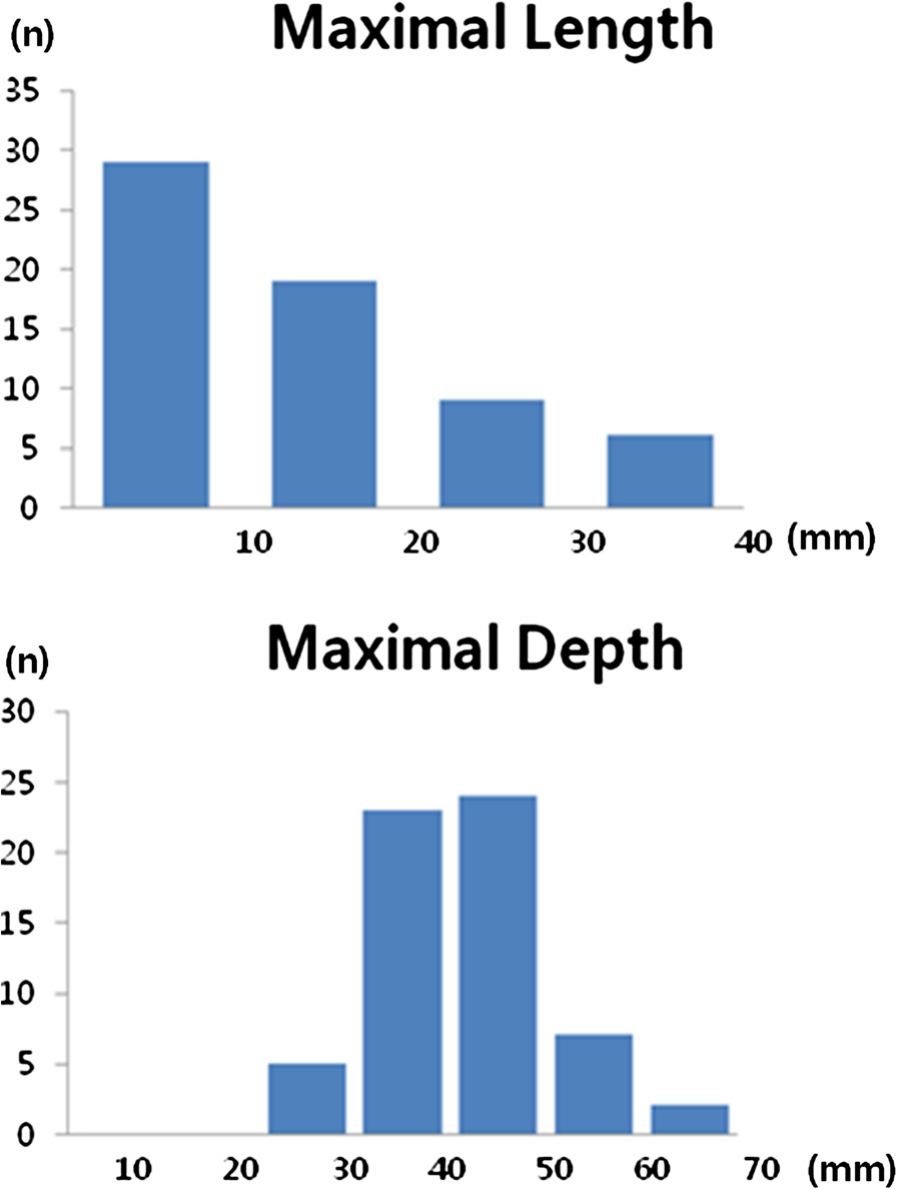
**Location of level II axillary lymph node. A**. The distribution of the maximal length from the axillary vessels to the level II lymph node. **B**. The distribution of the maximal depth from surface to level II lymph node.

The length from the lower border of the axillary vessels to the most inferior point of level II lymph node is represented in Figure 5. Forty six percent (29 of 63) of axillary lymph nodes were within 1 cm below the lower border of the axillary vessels and 76% (48 of 63) were within 2 cm, and 90% (57 of 63) within 3 cm. The maximum length from the lower border of the axillary vessels to the most inferior point of level II lymph node ranged from 1.4 cm from 3.5 cm (median, 1.4 cm).

The depth of level II lymph node was measured from the surface and is represented in Figure 5. Only 10% (6 of 63) of level II lymph nodes were within 3cm from the surface and 46% (29 of 63) were within 4 cm, and 84% (53 of 63) were within 5 cm. The maximum depth of the level II lymph nodes ranged from 2.3 cm from 6.5 cm (median, 4.2 cm).

The maximum depth of the level III lymph nodes ranged from 1.9 cm from 5.6 cm (median, 3.4 cm). The depth of the level III lymph nodes was within 3 cm in 31% (10 of 32), within 4 cm in 84% (27 of 32).

The maximum depth of the supraclavicular lymph nodes (SCLN) ranged from 1.4 cm from 6.8 cm (median, 3.8 cm). The depth of the SCLN was within 3 cm in 38% (8 of 21), within 4 cm in 71% (15 of 21). The depth of the SCLN and axillary lymph nodes was within ±1 cm in 48% (10 of 21) of patients. The axillary lymph nodes were located at ≥1 cm shallower or greater in 19% (4 of 21) and 33% (7 of 21) of patients, respectively.

### The dose to the axillary lymph nodes and normal organs

**Figure 6:**
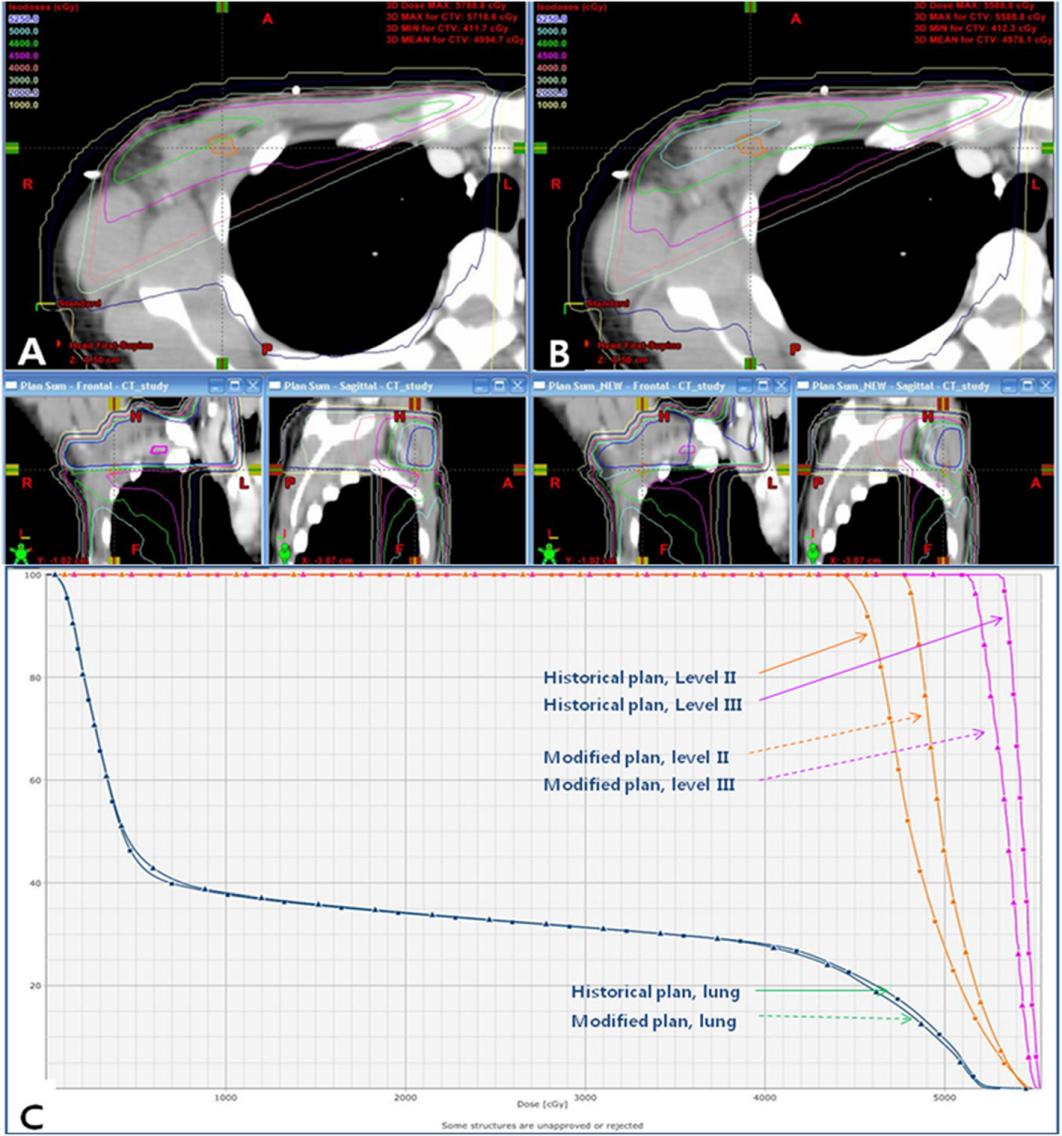
**Representative dose distribution and dose volume histogram (DVH) of axillary lymph nodes and ipsilateral lung in both plans. A**. Dose distribution in the historical plan at the level of level II lymph node. Orange colored area is initially involved level II lymph node area. Green line shows the area received 96% of the prescribed dose. **B**. Dose distribution in the modified plan. **C**. DVH of axillary LNs and ipsilateral lung in the two plans. The dose irradiated to the level II lymph node increased in the modified plan. The dose to level III lymph node in the modified plan was lower than in the historical plan but the doses were more than prescribed dose in both plans. The maximum dose decreased in the modified plan.

The differences in the dose distribution between the two plans are summarized in Table 4. Historically designed plan was not satisfactory with respect to covering the axillary level II lymph nodes. Only 57.7% of level II lymph nodes received more than 95% of prescribed dose. Level III lymph nodes received much higher dose than level II lymph nodes, so 96.8% of the volume received more than 95% of prescribed dose. Improvement in the coverage of level II lymph node was observed with the modified plan. The volume received more than 95% of prescribed dose (V95) increased from 57.7% to 70.0% statistically significantly (p < 0.01). Furthermore, the mean dose to level II lymph node increased. V95s for level III lymph node were similar in both plans (p = 0.35). Although mean dose to level III lymph node in the modified plan was lower than in the historical plan, the doses in the two plans were more than the prescribed dose. Maximum dose decreased in the modified plan compared to the historical plan (p < 0.01). Ipsilateral lung volumes received 5 Gy, 10 Gy and 20 Gy increased statistically significantly in the modified plan. Figure 6 shows representative dose distribution and dose volume histogram of axillary LNs and ipsilateral lung in both plans.

**Table 4:** Differences in the dose distribution between the two plans

	**Historical plan**	**Modified plan**	**P value**
**Maximum dose**	58.0 ± 1.33	56.4 ± 1.49	<0.01
**Level II**			
Mean D95	45.4 ± 3.3	46.7 ± 2.7	< 0.01
Mean V95	57.7 ± 36.9	70.0 ± 33.6	<0.01
Mean dose	48.3 ± 3.0	49.0 ± 2.2	<0.01
**Level III**			
Mean D95	51.3 ± 2.4	50.6 ± 2.1	<0.01
MeanV95	96.8 ± 14.7	97.9 ± 8.4	0.35
Mean dose	52.5 ± 1.9	51.9 ± 1.5	<0.01
**Lung**			
V5	38.6 ± 8.4	40.9 ± 8.3	<0.01
V10	31.4 ± 7.7	31.8 ± 7.7	<0.01
V20	27.8 ± 7.5	28.0 ± 7.5	<0.01
Mean dose	14.3 ± 3.4	14.3 ± 3.4	0.22

## Discussion

In the treatment of breast cancer, locoregional RT has been proven to improve overall survival in women with lymph node positive breast cancer [4-7]. Radiotherapy for breast cancer is usually given postoperatively, so RT technique is standardized irrespective of the initial location of the tumor. The existence of residual disease after initial treatment may lead to distant dissemination [3].

### The accuracy of the DIR

In this study, we used DSC and the distance between COMs to evaluate the performance of DIR. The DSC was low for axillary vessels. There are several reasons in low DSC. First, simulation CT for the radiotherapy was acquired with a sloped flat breast board, whereas PET-CT was acquired with a curved scanner table. The arm position was largely changed during the deformation. Second, the locations of axillary vessels might be changed after the removal of enlarged axillary lymph nodes around the axillary vessels at the time of surgery. Third, DSC is not very useful for very small volumes [18]. The volume of axillary vessels is so small that no overlap in a few slices results in a low DSC.

The distance between the COMs for structures showed similar pattern. The distance between the centers of axillary vessels was largest among the structures and it was prominent in lateral direction. Usually axillary vessels are present in three or four CT slices and vessels might be either longer or shorter according to the cutting angle. Some difference between the lengths of the axillary vessels in any CT slice can change the locations of centers of axillary vessels, especially in lateral direction, which can lead to the large value in the distance between the COMs.

The DSCs of bony structures were higher than 0.7. It was proposed by Zijdenbos et al. that a coefficient larger than 0.70 represents a good overlap [19]. This was accomplished in bony structures. The distance between the COMs for bony landmarks such as clavicle, coracoids process is relatively small but high in humeral head. It may be influenced by change of arm position between PET/CT and simulation CT. In consideration of good results of bony structures, the DIR of initially involved axillary lymph nodes is generally acceptable.

### PET-CT

PET-CT is a useful diagnostic tool for the evaluation of breast cancer, especially locally advanced breast cancer. PET or PET-CT have lower sensitivity and specificity than sentinel LN biopsy, therefore they cannot replace sentinel LN biopsy [8,9]. However, the positive predictive value of PET or PET/CT was 84%, so hypermetabolism of an axillary lymph node in noninfectious condition is highly suggestive of metastasis [8]. As Level III and supraclavicular LN area are not usually dissected in routine axillary LN dissection, it is very important to include these preoperatively detected LN areas in radiotherapy. In our study, 80% of patients were confirmed to have axillary lymph node metastasis preoperatively and 8% of patients were confirmed postoperatively. PET-CT image showing hypermetabolism in axillary lymph node suggests high possibility for the presence of a pathologic lymph node. Such nodal areas should be treated adequately to eradicate possible microscopic residual disease.

### The location and the coverage of axillary lymph node

The depth of axillary LN and SCLN was comparable to other study measuring depth of lymph nodes [20]. Bentel et al. showed the median maximum depth of axillary LN and SCLN were both 4.3 cm and the depth of axillary LN was less than 3 cm in 16% (8 of 49) of patients. The median maximum depth of level II axillary lymph node and SCLN was 4.1 cm and 3.8 cm, respectively in this study. And the depth of axillary level II LN was within 3 cm in 10% (6 of 63) in this study. However, the location of lymph nodes was estimated based on the location of the vessels, scar, or clips on simulation CT in Bentel’s study. It may be helpful to estimate more accurate initial location of lymph nodes to use DIR of preoperative PET/CT.

Recently published contouring atlas defined that caudal border of axillary level III LN is 1 cm caudal to the axillary vessels [21]. However, only 46% of axillary lymph nodes were within 1 cm below the lower border of the axillary vessels in this study. Therefore we expect that our data can be integrated into further guideline of contouring volumes.

When the junction between anterior beam and tangential beams is located at the inferior end of the clavicular head, lymph nodes near the junction receive lower dose than the prescribed dose. To overcome this underdose around the junction, the junction may be moved inferiorly than the lower end of the clavicular head, but it can result in overdose to the lung. Matchline shift during the course of treatment is the most common method to reduce underdose area, but there is a possible uncertainty and it is time-consuming. By using half-beam technique and modified anterior field weighted 10% of monitor unit, we could improve the coverage of level II lymph node without increasing maximum dose. We suggest that modified technique using modified anterior field weighted 10% of prescribed dose can be an alternative way to reduce underdose around the junction.

## Conclusion

Deformable image registration of PET-CT on simulation CT was acceptable in the identification of the location of the preoperatively involved axillary lymph node. Historically designed three field technique was not adequate to treat the axillary level II lymph node area. New treatment technique based on the location of axillary lymph node from PET-CT using DIR can result in more adequate coverage of nodal area.

## Abbreviations

DIR: Deformable image registration; MRM: Modified radical mastectomy; LAP: Lymphadenopathy; DSC: Dice similarity coefficient; COM: Center of mass; PAB: Posterior axillary boost; DRR: Digitally reconstructed radiography; RT: Radiotherapy; ALND: Axillary lymph node dissection; SCN: Supraclavicular lymph node; SCND: Supraclavicular lymph node dissection; DVH: Dose volume histogram.

## Competing interests

We declare that we have no significant competing financial, personal interest that might have influenced the performance or presentation of the work described in this manuscript.

## Authors’ contribution

L and K acquired the data and drafted the manuscript. Ahn participated in its design. C and K coordinated the study. L and S helped to draft the manuscript. Y and K carried out the statistical analysis. Park and Cho performed the deformable image registration. K conceived and directed the study. All authors read and approved the final manuscript.
